# Hospital Admission and Criminality Associated with Substance Misuse in Young Refugees – A Swedish National Cohort Study

**DOI:** 10.1371/journal.pone.0166066

**Published:** 2016-11-30

**Authors:** Hélio Manhica, Karl Gauffin, Ylva B. Almqvist, Mikael Rostila, Anders Hjern

**Affiliations:** Centre for Health Equity Studies (CHESS), Karolinska Institutet/Stockholm University, Stockholm, Sweden; Queensland University of Technology, AUSTRALIA

## Abstract

**Background:**

High rates of mental health problems have been described in young refugees, but few studies have been conducted on substance misuse. This study aimed to investigate the patterns of hospital care and criminality associated with substance misuse in refugees who settled in Sweden as teenagers.

**Methods:**

Gender stratified Cox regression models were used to estimate the risks of criminal convictions and hospital care associated with substance misuse from national Swedish data for 2005–2012. We focused on 22,992 accompanied and 5,686 unaccompanied refugees who were aged 13–19 years when they settled in Sweden and compared them with 1 million native Swedish youths from the same birth cohort.

**Results:**

The risks of criminal conviction associated with substance misuse increased with the length of residency in male refugees, after adjustment for age and domicile. The hazard ratios (HRs) were 5.21 (4.39–6.19) for unaccompanied and 3.85 (3.42–4.18) for accompanied refugees after more than 10 years of residency, compared with the native population. The risks were slightly lower for hospital care, at 2.88 (2.18–3.79) and 2.52(2.01–3.01) respectively. Risks were particularly pronounced for male refugees from the Horn of Africa and Iran. The risks for all male refugees decreased substantially when income was adjusted for. Young female refugees had similar risks to the general population.

**Conclusion:**

The risks of criminality and hospital care associated with substance misuse in young male refugees increased with time of residency in Sweden and were associated with a low level of income compared with the native Swedish population. Risks were similar in accompanied and unaccompanied refugees.

## Introduction

Studies of young refugees in Scandinavia have demonstrated a high risk of mental health problems shortly after resettlement, such as anxiety, PTSD, sleeping disturbance, etc.; which result from their psychosocial life context associated with their status as refugees. However, these risks tend to fade over time, as young refugees improved their living condition and their legal status[1, 2].

Population studies have confirmed that the mental health of the refugee population is worse than natives and other migrants; this higher vulnerability have been explained by the higher burden of war related traumas and constant uncertainties in their lives[3–5]. In contrast, economic and family migrants, a self-selected group have better mental health outcomes than native population[3]; however, this health advantage is more likely to deteriorate over time, as these migrants are more likely to end up in a low socio economic position[4].

Illicit drug misuse and drug related crime in young refugees has not been studied much in Scandinavia, but patterns found in youths with foreign-born parents suggest a high prevalence[6, 7]. Socioeconomic vulnerability in the destination country has been identified as a major pathway to drug related crime and hospital care due to substance misuse among immigrants in Scandinavia and in refugees in particular. This issue have been associated with factors such as long-term unemployment and living in low status neighborhoods, characterized by high levels of criminality, poor housing quality and deficient public services[6, 8–11].

Sweden has developed into a multicultural society as refugees and their families have dominated immigration to Sweden since the mid-1970s. The country has also received increasing numbers of unaccompanied refugee children, mostly male teenagers from Somalia, Afghanistan and Iraq[5, 12]. Unaccompanied refugees might be more vulnerable to misuse illicit substances as they are at higher risk of mental health problems compared with other youths of the same age[13]. Apart from the psychological distress they experienced in their country of origin[14],migrants and refugees are often exposed to important socio-environmental stressors in their destination countries that might lead to misuse of illicit substance. These include discrimination, long-term unemployment, low income and living in substandard housing in deprived socioeconomic neighbourhoods[5, 15]. In addition, we might expect gender differences in relation to criminality and hospital care associated with substance misuse as this has been documented in previous migrant studies conducted in Sweden and elsewhere[6, 16].

This study aimed to investigate patterns of criminality and hospital care associated with substance misuse among unaccompanied and accompanied youth refugees and their native Swedish peers. We considered the role of gender, socio-demographic context and income and investigated the extent to which country of origin and length of residence was associated with criminality and hospital care associated with substance misuse.

## Methods

The study was approved by the Regional Ethics Committee in Stockholm before any records were linked (decision no. 2013/811-31/5). All the data we used were anonymous and the researchers did not have access to any personal information that could identify individuals included in the dataset. According to Swedish data protection laws, administrative data is made available for specific research projects. Thus, we cannot share the data we used for this study with other researchers. We, however, are happy to answer any questions about the data used in this study and to share unpublished results.

Sweden has a long tradition of maintaining national registers that provide high-quality data on health and socioeconomic indicators. These registers are protected by special legislation, which makes it possible to collect certain data without the personal consent of individuals[17]. Different registers can be linked to each other by using the unique personal identification number that follows Swedish residents from birth or immigration to death. In this register-based study, we linked immigration and socio-demographic information from registers held by Statistics Sweden to health data held by the Swedish Migration Agency of Health and Welfare and The Register of Criminal Offenses held by the National Council for Crime Prevention.

### Study population

The study population comprised individuals who were born between 1972 and 1984 and were, according to the Register of the Total Population, alive and resident in Sweden on December 31 2004. We identified 27,688 individuals who were aged 13 to 19 years when they settled in Sweden as refugees or because they were related to a refugee. The information regarding the refugee population was acquired from STATIV, a longitudinal database for integration studies, which is held by Statistics Sweden and based on data from the Swedish Migration Agency. The definition of a refugee was based on Article 1 of the 1951 Geneva Convention Relating to the Status of Refugees and the 1967 Protocol. We also included refugees granted residence permits in Sweden on humanitarian grounds. The comparison population comprised 932,953 native Swedish born individuals with two Swedish-born parents.

### Main predictors

We used the Register of the Total Population to identify the largest sources of refugees to Sweden: Former Yugoslavian Republics (n = 9,438), Horn of Africa, including Somalia, Eritrea and Ethiopia (n = 3,408), Iraq (n = 4,671) Iran (n = 2,358) and others, including other non-European refugees (n = 7,813). The teenage refugees were categorised as accompanied young refugees (n = 22,002) if they had obtained residency because they were related to a family member who was a refugee, according to STATIV, or had at least one parent in the Multi-Generation register who had received residency in Sweden the same year or the year before the young refugee, according to the Register of the Total Population. On the other hand, young refugees who arrived alone, without the company of at least one parent (or an adult who by law or custom is responsible for their care) were classified as unaccompanied young refugees (n = 5,686). The length of residence for male refugees was categorised into: 0–5, 6–10 and 11 and more years on residence. Due to statistical power, the length of residence for refugee women was dichotomised as less than, or more than, 10 years, based on information on the year of immigration from the Register of the Total Population.

### Outcome

We created two outcomes of substance misuse. The first outcome was hospital care, which referred to hospitalisation due to substance misuse, with a diagnosis of substance use disorder according to the Swedish national inpatient and outpatient registers. The number of death due to substance misuse and the most common illicit substances leading to hospital care were cannabis, followed by opium, heroin and amphetamine. The second outcome was criminal conviction related to substance misuse; this was based on data from Swedish prosecutors and courts and from the National Police. This referred to: transferring, manufacturing, acquiring the for purpose of transfer, procuring and processing, packaging, transporting, keeping, offering for sale, possessing, using or otherwise handling narcotic drugs, according to the Swedish Penal Law on Narcotics (1968:64). Unfortunately, our dataset did not provide information regarding criminal convictions related to narcotic drugs.

### Covariates

Age, gender, disposable income and domicile were retrieved from the 2008 Longitudinal Integration Database for Health Insurance and Labor Market Studies. *Disposable income* was divided into quintiles, which included all registered sources of income after taxes and was then divided by the consumer units in the household, according to a formula developed by Statistics Sweden. The range of income in quintiles was given in Euros (Current rates from Swedish Krona to Euro in December 2004 ForexBank): quintile1 (11–8.970) quintile2 (8.971–12.455), quintile3 (12.456–16.661) quintile4 (16.662–22.158) and quintile5 (22.159–937.000). *Domicile* was split into three categories and defined by the place of residence in 2008: a “big city” referred to the metropolitan areas of Sweden’s three largest cities, Stockholm, Gothenburg and Malmo, a “town” covered other predominately urban communities and “rural” covered the remainder.

### Statistical analysis

The analyses were based on person-time measured from 1 January 2004 to whatever occurred first: death, the first recorded hospital admission to specialised psychiatric care due to substance misuse, a criminal offence or the end of follow-up period on 31 December 2012.

We first compared the incidence of hospital care and criminal convictions among unaccompanied and accompanied refugees with native Swedish peers as the reference group.

Cox proportional hazard models were estimated to compare the hazard ratios of hospital care due to substance misuse and criminal convictions among refugees with their native Swedish peers. We applied the method developed by Weitoft et al in 1999[18] in order to minimise any bias caused by unrecorded migration in our study population. For example, a year without any information on household income from labour or other benefits was considered to be an indicator of emigration.

We performed a gender stratified analysis of the two outcomes variables, hospital care and criminal conviction, between unaccompanied and accompanied refugees based on their length of residency. For male refugees, the variable length of residence was categorised into 0–5, 6–10 and 11 or more years. However, in order to improve the statistical power, female refugees were categorised into up to 10 years and more than 10 years of residence in Sweden. Each longitudinal model was analysed with adjustments for age and domicile (Model 1) and income (Model 2). We also performed an additional analysis that considered the effect of country and region of birth on the outcome variables, with native Swedish peers as the reference group. We use the same adjustment procedure presented above. All models were tested for proportional hazard assumption. This assumption was not violated.

## Results

Table 1 presents the socio-demographic characteristics of the study population included in the analyses. The majority of the teenage refugee population were male. Unaccompanied refugees were slightly older, than accompanied refugees in 2004. Young refugees were more likely to live in big cities, namely the metropolitan areas of Sweden’s three largest cities, Stockholm, Gothenburg and Malmo. There were considerable differences in terms of length of residence among the different nationalities and between the accompanied and unaccompanied refugees. About 40 percent of refugees of Iraq had a period of residence of 5 years. The distribution of disposable income in quintiles showed considerable difference in the distribution of income across the refugee population, with refugees from the Horn of Africa and Iraq being overrepresented among those with the lowest income.

**Table 1:** Socio-demographic indicators of the whole study population (n = 960,641).

Variables	Sweden	F. Yugoslavia		Horn of Africa		Iraq		Iran		Others	
		Acc.	Unacc.	Acc.	Unacc.	Acc.	Unacc.	Acc.	Unacc.	Acc.	Unacc.
	1,255,782	7,980	1,458	1,721	1,687	3,726	945	1,794	564	6,781	1, 032
	%	%	%	%	%	%	%	%	%	%	%
**Gender**											
Male	51.6	52.3	53.8	55.9	60.9	53.8	72.1	54.5	75.7	53.3	64.1
**Mean age**	26.1	25.2	27.1	25.4	28.1	23.3	24.9	26.7	29.5	26.1	27.3
**Residency (in years)**											
0–5	-	6.2	7.68	10.9	7.1	34.4	42.5	9.3	3.7	13.9	21.5
6–10	-	64.4	35.5	37.7	32.1	43.1	31.9	23.5	8.2	29.1	17.6
11 and more	-	29.4	56.9	51.2	60.8	22.5	25.6	67.2	88.1	57.0	60.9
**Domicile**											
Big city	44.3	49.6	51.4	73.8	77.4	60.5	62.2	68.9	71.1	61.1	58.4
Town	42.5	45.2	42.3	23.8	21.1	37.1	35.9	28.7	26.1	35.2	37.8
Rural	13.2	5.2	6.3	2.4	1.5	2.4	1.9	2.4	2.8	3.7	3.8
**Income**											
1	10.2	24.3	22.6	42.7	38.1	57.7	43.7	31.6	23.3	41.0	39.6
2	16.7	24.5	25.8	22.2	23.5	20.0	22.9	21.6	17.0	23.2	23.7
3	21.2	22.6	22.1	12.9	13.2	11.0	13.1	16.9	18.3	15.5	15.1
4	24.9	18.8	16.7	13.4	14.0	7.2	11.8	14.6	17.6	12.7	12.7
5	27.0	9.6	12.8	8.7	11.3	4.1	8.5	15.3	23.8	7.6	8.9

Acc. = accompanied; Unacc. = unaccompanied; Sweden refers to native Swedish population; other countries = all non-European refugees

The incidence of criminal convictions and hospital care associated with substance misuse are presented in Table 2. The table shows that the rate of criminal convictions for male refugees was about three times higher than for native Swedish males. In contrast, the rates were lower in female refugees compared to native Swedish females. The rates of hospital care for male refugees were higher among unaccompanied refugees compared with Swedish males. These rates were lower in female refugees compared with Swedish females.

**Table 2:** A. Incidence of criminal convictions and hospital care due to substance misuse in male and female refugees and Swedish-born peers (n = 960,641).

Variables	Criminal convictions				Hospital care due do substance misuse			
	Male		Female		Male		Female	
	Cases	Incidence rate	Cases	Incidence rate	Cases	Incidence rate	Cases	Incidence rate
		(95% CI)		(95% CI)		(95% CI)		(95% CI)
**Study population**								
Sweden	10,464	273.7 (271–282)	1,933	53.6 (52–56)	6,366	167.3 (163–171)	2,818	78.2 (75–81)
Unaccompanied refugees	232	846.4 (744–962)	7	41.8 (20–87)	87	250.4 (250–301)	10	59.7 (32–111)
Accompanied refugees	842	943.9 (882–1009)	25	30 (20–45)	310	336.7 (301–376)	43	52.4 (39–70)
**Income (quintile)**								
1	3,247	859.8 (830–889)	666	153 (141–165)	1,827	472.8 (451–495)	767	176.2 (164–189)
2	2,149	392.6 (376–409)	554	70.1 (70–82)	1,392	252.4 (239–266)	767	105.6 (98–113)
3	2,148	300 (287–313)	381	43 (39–47)	1,406	195.2 (185–205)	672	76.3 (70–82)
4	2,145	230 (221–240)	247	26.3 (23–29)	1,216	130.1 (122–137)	457	48.8 (44–53)
5	1,633	124 (118–130)	99	13.9 (11–17)	805	65.2 (60–69)	194	27.3 (23–31)
**Origen**								
F.Yugoslavia	270	710.9 (630–800)	7	19.9 (9–40)	101	259.7 (213–315)	17	47.3 (29–76)
Horn of Africa	219	1484 (1300–1694)	9	79.9 (41–153)	60	384.9 (298–495)	7	62.1 (29–130)
Iraq	144	694.6 (589–817)	4	25.2 (9–67)	57	269.3 (207–349)	8	50.5 (25–101)
Iran	90	838 (682–1031)	5	65.8 (27–158)	60	550.6 (427–709)	12	158.2 (89–278)
Other	351	1082 (975–1202)	7	24.9 (11–52)	119	353.4 (295–423)	9	31.9 (16–61)
**Domicile**								
Big city	5,149	304.7 (296–313)	805	48.7 (45–52)	2,930	172.2 (172–179)	1,268	76.8 (72–81)
Town	4,739	289.3 (281–297)	888	58.4 (54–62)	2,832	171.8 (165–178)	1,209	79.8 (75–84)
Rural	1,322	262.9 (249–277)	230	43.9 (43–56)	788	155.8 (145–167)	349	75.9 (68–84)
**Residency (years)**								
0–5 years	164	982.5 (843–1145)	8	57.2 (28–114)	47	270 (203–360)	25	59.7 (40–88)
6–10 years	483	1038 (949–1135)	8	18.9 (9–38)	167	346 (297–403)	20	46.6 (30–72)
11 and more	427	799.6 (727–879)	16	37.1 (22–60)	183	335 (289–387)	24	37.4 (37–83)

Incidence rate per 100,000 person years

The results of Table 2 are also reflected in Table 3 including the gender stratified Cox regression analysis. Table 3 shows that male refugees were at higher risks of hospital admission and criminality associated with substance misuse with native Swedish males. A longer period of residence was associated with increased risks of criminal conviction and hospital care associated with substance misuse in male refugees. In fact, the hazard ratios (HRs) of criminal conviction were 1.35 (0.92–1.99) in unaccompanied and 2.44 (2.06–2.90) in accompanied refugees with a period of residence of up to five years. These risks increased to 5.21 (4.39–6.19) and 3.85 (3.42–4.18), respectively, when the period of residence was longer than 10 years. In addition, a period of residence of more than 10 years was also associated with increased HRs of hospital care among unaccompanied and accompanied refugees compared with native Swedish when age and domicile were adjusted for, of 2.88 (2.18–3.79) and 2.52 (2.01–3.01) respectively. These risks were further attenuated when income was adjusted for. The results for the female population were more difficult to interpret due to wider confidence intervals, but in most cases the accompanied and unaccompanied females refugees had lower risks for criminal convictions and hospital care than their Swedish peers.

**Table 3:** Cox regression models for hospital care and criminality associated with substance misuse among unaccompanied and accompanied refugees by length of residency and gender (n = 960,641).

Gender	Study population by years of residency	Criminal convictions		Hospital care	
		HR 95% CI	HR 95% CI	HR 95% CI	HR 95% CI
		Model1	Model2	Model1	Model2
**Men**	Native Swedish	1	1	1	1
	Unaccompanied refugees: residency (0–5)	1.35 (0.92–1.99)	0.88 (0.60–1.30)	0.66 (0.31–1.38)	0.42 (0.20–1.88)
	Unaccompanied refugees: residency (6–10)	3.86 (3.02–4.94)	2.65 (2.07–3.41)	2.47 (1.68–3.63)	1.60 (1.03–2.37)
	Unaccompanied refugees: residency (11+)	5.21 (4.39–6.19)	3.44 (2.87–4.11)	2.88 (2.18–3.79)	1.95 (1.48–2.57)
	Accompanied refugees: residency (0–5)	2.44 (2.06–2.90)	1.33 (1.12–1.59)	1.25 (0.90–1.72)	0.66 (0.47–0.91)
	Accompanied refugees: residency (6–10)	3.22 (2.92–3.56)	2.13 (0.93–2.36)	1.81 (1.53–2.14)	1.17 (1.00–1.39)
	Accompanied refugees: residency (11+)	3.85 (3.42–4.18)	2.55 (2.25–2.89)	2.52 (2.01–3.01)	1.65 (1.37–1.97)
**Women**	Native Swedish	1	1	1	1
	Unaccompanied refugees: residency (0–10)	0.46 (0.11–1.84)	0.25 (0.06–1.03)	0.64 (0.24–1.73)	0.40 (0.15–1.09)
	Unaccompanied refugees: residency (11+)	1.80 (0.74–4.34)	1.11 (0.46–2.52)	1.18 (0.53–2.64)	0.80 (0.36–1.80)
	Accompanied refugees: residency (0–10)	0.43 (0.25–0.73)	0.25 (0.15–0.34)	0.57 (0.38–0.85)	0.38 (0.26–0.56)
	Accompanied refugees: residency (11+)	0.81 (0.43–1.51)	0.46 (0.24–0.87)	0.85 (0.53–1.35)	0.59 (0.37–0.94)

Model 1 adjusted for age and domicile Model 2 adjusted for age, domicile and income. HR = hazard ratio, 95%CI = confidence intervals

Table 4 shows Cox regression models of hospital care and criminality associated with substance misuse by specific country of origin. Compared with native Swedish males, male refugees from the Horn of Africa and Iran had the highest HRs of criminal convictions and hospital care. These risks decreased when age, domicile and income were adjusted for, particularly in refugees from the Horn of Africa. The risks of hospital care and criminality due to substance misuse were generally lower in female refugees than native Swedish women. There was an exception in the HRs of female refugees from Iran, who received hospital care; HR = 2.27 (1.29–3.40), but this risk disappeared in the fully adjusted model.

**Table 4:** Cox regression models for hospital care and criminality associated with substance misuse, by country of origin and gender (n = 960,651).

Gender	Swedish population	Criminal convictions		Hospital care	
		HR 95% CI	HR 95% CI	HR 95% CI	HR 95% CI
		Model 1	Model 2	Model 1	Model 2
**Men**	Swedish population	1	1	1	1
	F. Yugoslavia	2.50 (2.21–2.82)	1.86 (1.64–2.11)	1.46 (1.19–1.79)	1.07 (0.88–1.32)
	Horn of Africa	6.67 (5.82–7.63)	4.19 (3.64–4.82)	2.84 (2.20–3.67)	1.77 (1.37–2.30)
	Iraq	1.94 (1.64–2.30)	1.09 (0.92–1.29)	1.35 (1.03–1.76)	0.73 (0.56–0.97)
	Iran	3.72 (3.02–4.60)	2.44 (1.97–3.01)	3.81 (2.49–4.93)	2.51 (1.93–3.25)
	Other	4.11 (3.70–4.58)	2.47 (2.21–2.76)	2.21 (1.84–2.66)	1.30 (1.08–1.57)
**Women**	Swedish population	1	1	1	1
	F. Yugoslavia	0.34 (0.17–0.76)	0.25 (0.12–0.53)	0.59 (0.37–0.96)	0.45 (0.28–0.73)
	Horn of Africa	1.67 (0.83–3.34)	0.95 (0.47–1.92)	0.97 (0.44–1.97)	0.61 (0.29–1.12)
	Iraq	0.37 (0.14–1.00)	0.18 (0.07–0.50)	0.56 (0.28–1.12)	0.32 (0.16–0.65)
	Iran	1.48 (0.61–3.56)	0.94 (0.39–2.26)	2.27 (1.29–3.40)	1.63 (0.92–2.86)
	Other	0.52 (0.24–1.08)	0.24 (0.11–0.55)	0.44 (0.22–0.84)	0.27 (0.14–0.53)

Model 1 adjusted for age and domicile; Model 2 adjusted for age, domicile and income. HR = hazard ratio, 95% CI = 95% confidence intervals

## Discussion

This register follow-up study of hospital care and criminality associated with substance misuse in young refugees who settled in Sweden as teenagers suggests that accompanied and unaccompanied male refugees had higher risks of hospital care and criminality associated related with substance misuse than native Swedish males, while the opposite pattern was found among female refugees. The longer that male refugees had resided in Sweden, the more the risks of hospital care and criminality related with substance misuse increased. Refugees from the Horn of Africa and Iran were more likely to be convicted for a substance related offence and undergo hospital care for substance misuse, but these risks decreased when income was adjusted for.

Our findings that the risks of hospital care and criminal conviction related with substance misuse in the refugee population decreased considerably when they were adjusted for income suggests that it is important to consider the adverse living conditions faced by refugees in Sweden when interpreting the results. It has been shown that compared with the native Swedish population, refugees and migrants are likely to have lower income and live in segregated low-status neighborhoods[19, 20] where there is more access to illicit substances[21, 22]. Thus, it is possible that residents of socially disadvantaged neighborhoods might be more exposed to drug activities due to the higher presence of drug dealers and high society interactions with drug users[21, 23]. Another possible explanation for the higher risk of criminal convictions in male refugees could be the high presence of police patrols in these ethnically segregated neighborhoods, resulting in high numbers of drug arrests[24]. In the US, for example, studies have reported that ethnic and racial stereotypes within the police system might shape perception of crime; hence, police officers and offenders are believed to perceive non-white individuals as being more involved in different forms of crime activities. This hypothesis is justified by the higher concentration of police resources in neighborhood with high ethnic density[25]. These findings from the US are in line with studies conducted in Sweden that provided evidences of institutional discrimination within the police system, causing ethnic disparities in criminal justice outcomes[26, 27].

We could also speculate that the psychological distress experienced by refugees as a result of the traumatic experiences of war and separation from their families and friends, the migration and asylum process, discrimination, economic worries and acculturation challenges[5, 28–30] could contribute to increased risk of needing hospital care due to substance misuse. This is in line with previous studies that highlighted that refugees might use illicit substances for self-medication and as a coping strategy to deal with their stressful life experiences[31–33]. However, our study does not confirm the low patterns of substance use disorder found in refugees and other migrants in the US[34].

This study also reports higher rates of hospital care and criminal conviction associated with substance misuse in certain male refugee populations, including those from Iran and the Horn of Africa. These findings might reflect the patterns of drug use that refugees bring from their home countries, as found in previous studies[35]. In the Netherlands, for example, higher use of opiates and heroin were reported in the Iranian community, as well as khat, a fresh leafy plant that is chewed, among East Africans. The use of khat is not considered an addictive substance among East African people [31]. Paradoxically, opium has been associated with severe mental health problems in Iran although its use is socially accepted[36].

The observed discrepancy between the higher risk of criminal convictions and the more moderate risks of hospital care due to substance misuse suggests that many refugees might face significant barriers to seeking treatment for substance abuse. This is in line with a Swedish register-based study that found lower rates of psychiatric care usage among young refugees in Sweden (Manhica et al, in press)[37], suggesting that young refugees might face formal and informal barriers to accessing psychiatric care. Such barriers could include knowledge about the healthcare system, feelings of shame and stigma, low health literacy level, cultural factors affecting self-perceived needs and the use of healthcare[38–41]. Another potential explanation behind this discrepancy is that a substance related offence committed by an immigrant could be more likely lead to arrest and conviction than a similar offence committed by a native Swedish person.[42, 43]

The fact that the risks of hospital care due to substance misuse increased with the length of residence could be due to the refugees’ increased acculturation with Swedish values in relation to mental health-seeking behavior, as suggested by previous studies on mental healthcare in migrants[44]. In addition, it is possible that long-term social maladjustment and subsequent psychiatric problems could encourage misuse of illicit substance[45]. We could also speculate that it is probable that increased misuse of illicit substance in this group might lead to greater damage over time, resulting in an increased risk of needing hospital care. Further research is needed to clarify the role of social maladjustment on mental health and illicit substance misuse in refugee populations in Sweden.

With regard to gender differences, the results of the current study confirm previous findings that have repeatedly reported higher rates of substance use disorder in males than females[46, 47], suggesting that males are more likely to engage in drug activities[48]. Our study showed that female refugees had lower risks of hospital care and criminality associated with substance misuse than native Swedish females, suggesting that there might be cultural factors affecting the misuse of illicit substance and need for psychiatric care. However, further research is needed to clarify these mechanisms.

The major strength of our study was that it was based on national data, from a combination of different national registers covering the entire population living in Sweden during the follow-up period. We used the unique personal number allocated to all Swedish citizens to link the socio-demographic variables with data on hospitalisation due to substance misuse and criminal convictions. Our dataset allowed us to identify the refugee population living in Sweden as a result of the Geneva Convention, together with refugees granted residence permits on humanitarian grounds. This data also allowed us to identify their country of origin, as well as whether these refugees were accompanied or unaccompanied when they came to Sweden. The dataset provided information regarding access to healthcare and criminal convictions and this information was unique in itself, as it enabled us to capture cultural differences in health-seeking behaviours related to substance misuse and contrast it with data on criminal convictions.

This study also had some limitations. First, we could not estimate if the observed variations in the use of psychiatric care by country of origin reflected different health-seeking behaviours and barriers to care. Second, our data do not offer information related to the type of substance related to the criminal convictions or a direct indication of substance misuse indicator. In addition, one should also be aware that the outcome measuring criminality associated with substance misuse lumps together: possession, use, transportation and selling of any type of illegal substances. Third, the “others” and “East Africa” category used in the country of birth variable contained important population heterogeneity that should be considered when interpreting the results. The small population size for individual countries did not allow us to split these categories into smaller units.

In addition, information on immigration and length of residency was based on the date when a residence permit was granted, not the date when the person entered Sweden. Thus, it is possible that some refugees could have lived in Sweden for longer than estimated. Essential data on the mechanisms underlying the associations were not available in our data, such as information about cultural values, neighbourhood characteristics, social support or different types of ethnic discrimination that could account for the different patterns in the outcome variable.

## Conclusion

Unaccompanied and accompanied male refugees are at particular risks of hospital care and criminal convictions associated with substance misuse in Sweden and this should be highlighted in prevention and healthcare policies that are sensitive to the geographical, linguistic and cultural backgrounds of this migrant population. These higher risks were associated with a lower level of income. Further studies are needed to elucidate the pathways between low income and the increased risks of hospital care and criminality associated with substance misuse with time of residency. These studies would support policy makers when they develop health policies linked to social inequalities, as well as knowledge about how social inequalities influence health-related behaviours among vulnerable refugee groups.
